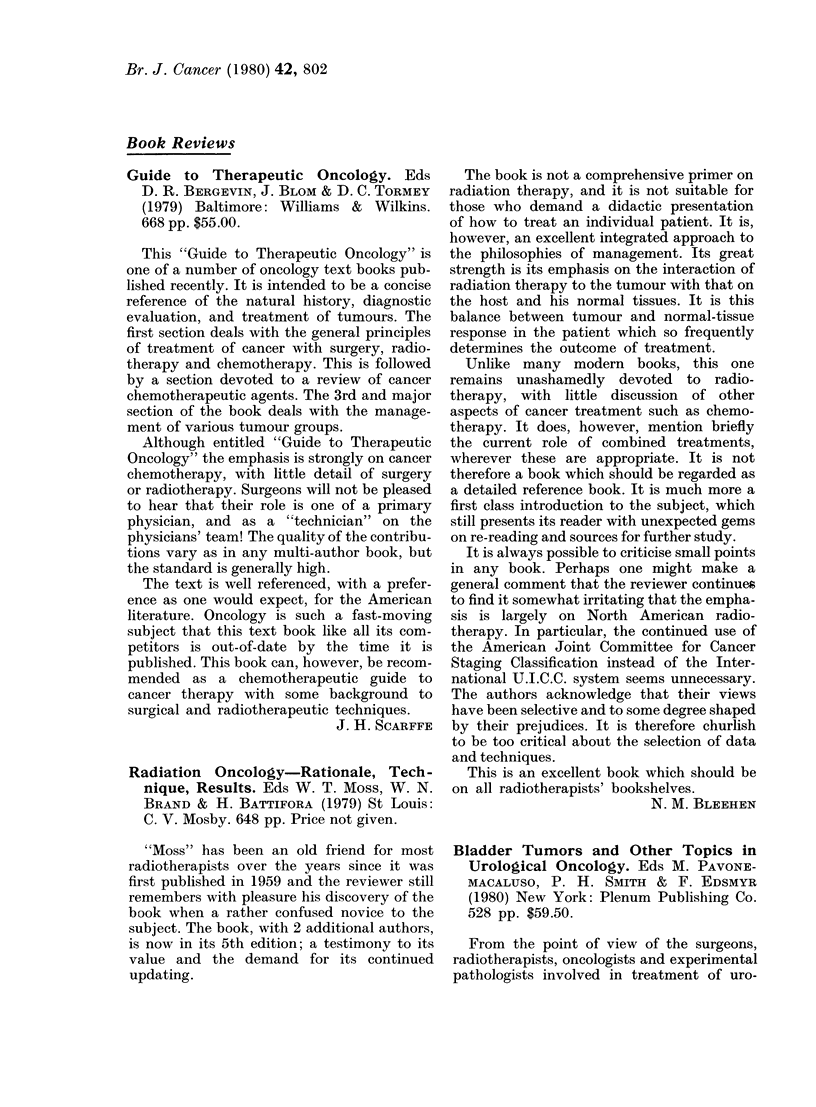# Radiation Oncology—Rationale, Technique, Results

**Published:** 1980-11

**Authors:** N. M. Bleehen


**Radiation Oncology—Rationale, Technique, Results.** Eds W. T. Moss, W. N. Brand & H. Battifora (1979) St Louis: C. V. Mosby. 648 pp. Price not given.

"Moss" has been an old friend for most radiotherapists over the years since it was first published in 1959 and the reviewer still remembers with pleasure his discovery of the book when a rather confused novice to the subject. The book, with 2 additional authors, is now in its 5th edition; a testimony to its value and the demand for its continued updating.

The book is not a comprehensive primer on radiation therapy, and it is not suitable for those who demand a didactic presentation of how to treat an individual patient. It is, however, an excellent integrated approach to the philosophies of management. Its great strength is its emphasis on the interaction of radiation therapy to the tumour with that on the host and his normal tissues. It is this balance between tumour and normal-tissue response in the patient which so frequently determines the outcome of treatment.

Unlike many modern books, this one remains unashamedly devoted to radiotherapy, with little discussion of other aspects of cancer treatment such as chemotherapy. It does, however, mention briefly the current role of combined treatments, wherever these are appropriate. It is not therefore a book which should be regarded as a detailed reference book. It is much more a first class introduction to the subject, which still presents its reader with unexpected gems on re-reading and sources for further study.

It is always possible to criticise small points in any book. Perhaps one might make a general comment that the reviewer continues to find it somewhat irritating that the emphasis is largely on North American radiotherapy. In particular, the continued use of the American Joint Committee for Cancer Staging Classification instead of the International U.I.C.C. system seems unnecessary. The authors acknowledge that their views have been selective and to some degree shaped by their prejudices. It is therefore churlish to be too critical about the selection of data and techniques.

This is an excellent book which should be on all radiotherapists' bookshelves.

N. M. Bleehen